# Whole body tracking of superparamagnetic iron oxide nanoparticle-labelled cells – a rheumatoid arthritis mouse model

**DOI:** 10.1186/scrt337

**Published:** 2013-10-17

**Authors:** Hareklea Markides, Oksana Kehoe, Robert H Morris, Alicia J El Haj

**Affiliations:** 1 Institute for Science and Technology in Medicine, Keele University, Staffordshire, UK; 2 Institute for Science and Technology in Medicine at RJAH Orthopaedic Hospital Keele, Shropshire, UK; 3 School of Science and Technology, Nottingham Trent University, Nottingham, UK

## Abstract

**Introduction:**

The application of mesenchymal stem cells (MSCs) in treating rheumatoid arthritis (RA) has been made possible by the immunosuppressive and differentiation abilities of these cells. A non-invasive means of assessing cell integration and bio-distribution is fundamental in evaluating the risks and success of this therapy, thereby enabling clinical translation. This paper defines the use of superparamagnetic iron oxide nanoparticles (SPIONs) in conjunction with magnetic resonance imaging (MRI) to image and track MSCs *in vivo* within a murine model of RA.

**Methods:**

Murine MSCs (mMSCs) were isolated, expanded and labelled with SiMAG, a commercially available particle. *In vitro* MRI visibility thresholds were investigated by labelling mMSCs with SiMAG with concentrations ranging from 0 to 10 μg/ml and resuspending varying cell doses (10^3^ to 5 × 10^5^ cells) in 2 mg/ml collagen prior to MR-imaging. Similarly, *in vivo* detection thresholds were identified by implanting 3 × 10^5^ mMSCs labelled with 0 to 10 μg/ml SiMAG within the synovial cavity of a mouse and MR-imaging. Upon RA induction, 300,000 mMSCs labelled with SiMAG (10 μg/ml) were implanted via intra-articular injection and joint swelling monitored as an indication of RA development over seven days. Furthermore, the effect of SiMAG on cell viability, proliferation and differentiation was investigated.

**Results:**

A minimum particle concentration of 1 μg/ml (300,000 cells) and cell dose of 100,000 cells (5 and 10 μg/ml) were identified as the *in vitro* MRI detection threshold. Cell viability, proliferation and differentiation capabilities were not affected, with labelled populations undergoing successful differentiation down osteogenic and adipogenic lineages. A significant decrease (P < 0.01) in joint swelling was measured in groups containing SiMAG-labelled and unlabelled mMSCs implying that the presence of SPIONs does not affect the immunomodulating properties of the cells. *In vivo* MRI scans demonstrated good contrast and the identification of SiMAG-labelled populations within the synovial joint up to 7 days post implantation. This was further confirmed using histological analysis.

**Conclusions:**

We have been able to monitor and track the migration of stem cell populations within the rheumatic joint in a non-invasive manner. This manuscript goes further to highlight the key characteristics (biocompatible and the ability to create significant contrast at realistic doses within a clinical relevant system) demonstrated by SiMAG that should be incorporated into the design of a new clinically approved tracking agent.

## Introduction

Current tissue engineering approaches focusing on restoring and regenerating articular cartilage damage are limited to the damage caused by trauma and osteoarthritis [1]. The chronic inflammatory environment of the rheumatic arthritic joint renders these techniques ineffective, as similarly to the original native cartilage, newly formed cartilage will again undergo destruction within the hostile environment [1]. Rheumatoid arthritis (RA, a chronic autoimmune disease) is characterised by pain, stiffness and inflammation of the synovial joint [1-3]. This results in the destruction of articular cartilage and affects approximately 1% of the global population [1,2,4,5]. Current RA treatments involve a combination of drug regimens to alleviate symptoms, such as pain and inflammation, while preserving joint function and maintaining quality of life [1,5]. Few patients have experienced complete drug free remission with little progress being made in restoring joint function and regenerating cartilage [1,5,6].

Advances in tissue engineering have emphasised the role of mesenchymal stem cells (MSCs) in treating autoimmune diseases, such as RA [1,2,7]. Their specific self-renewal, multipotent differentiation ability (osteoblasts, chondrocytes and adipocytes), migratory, anti-inflammatory and immunosuppresssive properties are all key characteristics linked to their success in stem cell-based therapies [1,2,8-10]. These are modulated by the secretion of bioactive molecules. The immunosuppressive properties of MSCs are of particular importance in treating autoimmune diseases, such as RA [1]. The release of cytokines and growth factors, such as IL-10, IL-6, IL-11 and transforming growth factor – β (TGF-β), acts to inhibit T cells and dendritic cells [7,11] while the secretion of soluble antigens, such as human leukocyte antigen G (HLA-G), effectively disables natural killers and moderate dendritic cell and T cell activity. In addition, secreted immunosuppressive enzymes, such as indoleamine 2, 3-dioxygenase (IDO), suppress leukocytes such as B cells [7,11]. The combined secretion of these factors, their role in tissue homeostasis and repair (governed by a signalling mechanism) [2] and the cartilage forming ability of MSCs provides a trophic regenerative environment, stimulating the proliferation and differentiation of tissues to achieve intrinsic repair while protecting the neo tissue in a localised immunosuppressive manner [1,7,11].

Very little is known of the *in vivo* events occurring post implantation. A means of imaging and tracking implanted MSCs could prove extremely valuable in evaluating and optimising mechanisms of cartilage repair within an inflammatory environment. Information linked to cell migration [12], rate of repair [12] and tissue integration are pivotal in optimising the therapy in terms of cell number [13], cell dose [12], dosage schemes [14] and delivery methods [12]. Traditional means of gathering such data have relied on histological tests on sacrificed animals [15-17]. This tends to be invasive and information limited. The shortcomings of these techniques render them unacceptable in assessing the success of the cellular therapies [15-17].

In response to this, superparamagnetic iron oxide nanoparticles (SPIONs) can be employed in conjunction with the use of magnetic resonance imaging (MRI) to track implanted cells *in vivo*[16,18,19]. To date, little work has been carried out to optimise SPION concentrations for specific tissue applications *in vivo*. To this end, we have optimised towards the current clinical MRI modalities commonly found in hospitals, these being 1.5 tesla (T) and 3 T scanners. In essence, stem cells are encouraged to internalise SPIONs; through this method, the magnetic properties of the particles are transferred to the cells. The intracellular iron disturbs the local magnetic field thus allowing cells to be visualised as a lack of signal with MRI [19-25].

Here we have adopted a murine model of RA to investigate the immunomodulating and anti-inflammatory properties of mouse MSCs (mMSCs) labelled with and without SPIONs and to optimise the *in vivo* imaging and tracking protocol. To our knowledge, this is the first time MSCs have been tracked using SPIONS within a rheumatic joint in this manner.

## Materials and methods

### Mice

Experiments were undertaken in either 7- to 10-week-old inbred C57Bl/6 or BALB/c mice as specified (Harlan, Bicester UK). Procedures were performed in accordance with Home Office-approved project licence PPL 40/3594 and were approved by the Ethical Review Committee at Liverpool John Moores University UK, February 2012.

### Cells

mMSCs were isolated from BALB/c mice as previously described [26]. In brief, femurs and tibias were removed and flushed to isolate bone marrow cells. Cells were subsequently plated and incubated in cell isolation media (CIM) (RPMI-1640; Lonza, Slough UK) supplemented with 9% fetal bovine serum (FBS; Lonza, Biowhittakar), 9% horse serum, 1% penicillin-streptomycin (Gibco, Paisley UK) and cultured under standard conditions for 24 hours. Non-adherent cells were then removed and four weeks later cells re-plated at a seeding density of 100 cells per cm^2^ in complete expansion media (CEM; Iscove Modified Dulbecco Medium (IMDM); Invitrogen, Paisley UK) supplemented with 9% FBS, 9% horse serum and 1% penicillin-streptomycin for MSC expansion.

### Cell labelling

#### SPIONS

mMSCs were labelled with SiMAG (***a commercially available particle, 1,000*** nm particle size) (Chemicell, Berlin Germay). These particles have an unmodified silica surface with terminal negatively charged silanol groups. Cells were labelled at either passage (P) 12 or 13 using a 24 hour passive incubation method in serum free - CIM. Following incubation, 3 × PBS washes were performed in order to remove excess particles attached to the surface of the cells and flask.

#### CM-DiI labelling

Briefly, a stock solution of the fluorescent cell-tracer CM-DiI (Molecular Probes, Paisley UK) was prepared in dimethyl sulfoxide (DMSO) at a concentration of 1 mg/ml. MSCs were trypsinised, washed with PBS and incubated in the working solution of CM-DiI for five minutes at 37°C, and then for an additional 15 minutes at 4°C, in the dark. Unincorporated dye was removed via centrifugation (300 g for five minutes) and washed with PBS before resuspending in serum-free IMDM and maintained at 4°C until injection.

### Cell characterisation

#### Differentiation

SiMAG-labelled MSCs were tested for their ability to undergo differentiation into osteocytes and adipocytes. Labelled cells were incubated (37°C, 5% CO_2_) for a period of 21 days in the relevant differentiation media. *Adipogenic media*: CEM supplemented with Insulin, Transferrin, Selenium Premix (ITS), 1:100, Sigma-Aldrich, Dorset, UK), dexamethesome (10^-6^ M), 3-isobutyl-1-methylaxanithine (0.5 μM, Sigma – Aldrich, Dorset UK) and indomethacin (100 μM, Sigma Aldrich). *Osteogenic media*; CEM supplemented with dexamethesome (10^-8^ M, Sigma – Aldrich), β – glycerophosphate (10 mM, Sigma – Aldrich) and ascorbate – 2- phosphate (88 ng/ml, Sigma – Aldrich). After three weeks of culture in differentiation media, cells were fixed (95% methanol, 15 minutes) and stained with the relevant histological dyes including: Oil Red O (stain for lipids) prepared using 0.18% Oil red ‘O’ (Sigma –Aldrich) prepared in 10% isopropyl alcohol (IPA). Calcium deposition was confirmed by an Alizarin red dye prepared using 1% Alizarin Red solution (Sigma – Aldrich) (pH 4).

#### Flow cytometry analysis (fluorescence activated cell sorting)

Murine MSCs, labelled with or without SiMAG were analysed for membrane receptor expression. Antibodies used in this study were as follows: anti-mouse CD31 (PECAM-1) PE, anti-human/mouse CD44 PE, anti-mouse CD11b PE, anti-mouse CD45 PE, anti-mouse CD105 PE, anti-mouse Ly-6A(Sca-1)PE (all from eBioscience Hatfield UK). Cells were incubated at 4°C for 30 minutes with the relevant antibody. As a negative control, cells were incubated with the isotype antibodies. Propidium iodide staining was included in the immunophenotyping to evaluate the viability of the cells. A minimum of 10,000 events were recorded for each analysis, using a FACScan flow cytometer and analysed using CellQuestPro software (Becton Dickinson, Oxford, UK).

### Viability and proliferation

#### MTT

The effect of particle labeling on the proliferation rate of mMSCs was investigated using the MTT assay (a tetrazolium salt; Sigma). The MTT assay specifically determines cell number as a function of mitochondrial activity. SiMAG-labelled mMSCs (10 μg/ml) were allowed to expand up to 24 hours and 7 days, after which point, 0.5 mg/ml MTT solution was added to each sample and incubated for 4 hours at 37°C. The MTT solution was removed and DMSO added to solubilise the MTT crystals for a further 10 minutes at 37°C. Negative control groups were treated with DMSO for 5 minutes. Aliquots (200 μl) of the resultant assay solution were transferred to a 96-well plate and absorbance (570/690; excitation/emission) was read using the Syngery 2 Biotek plate reader. Sample repeats were n = 3.

#### Live dead assessment

Cell viability was investigated using live/dead assay. SiMAG-labelled mMSCs (10 μl/ml) were allowed to expand up to 24 hours and 7 days before being treated with 1% calcein AM and 2% propidium iodide prepared in PBS according to the manufacturer’s instructions for 45 minutes at 37°C, whilst protected from light. Samples were imaged using a UV fluorescent microscope (Nikon Eclipse Ti-S).

### Quantification of particle uptake

#### Prussian blue

mMSCs labeled with SIMAG (10 μg/ml) were fixed and permeabilised using 95% methanol for 15 minutes at room temperature. Cells were then treated with a solution made of a 1:1 ratio of 20% aqueous solution of HCl and 10% aqueous solution of potassium ferrocyanide (20 minutes, room temperature) (n = 3). Cells were imaged with a light microscope (Nikon – Eclipse TS 100).

#### Inductively coupled plasma optical emission spectrometry

Inductively coupled plasma optical emission spectrometry (ICP - OEAS) was performed to quantify the uptake of particles by mMSCs. A total of 300,000 cells were labelled with SiMAG (10 μg/ml) (n = 3). Labelled cells were collected and immersed in 1 ml of concentrated analytical grade nitric acid and heated to 60°C overnight for the degradation of particles in order to release Fe content. Samples were then diluted with dH_2_0 to achieve a final acid concentration of less than 10% prior to analysis using ICP.

### Induction of murine antigen-induced arthritis

Experiments were performed in seven- to eight-week-old male C57B1 mice. Murine antigen-induced arthritis (AIA) was induced as described [27]. Briefly, mice were immunised subcutaneously with 1 mg/ml of methylated BSA emulsified with an equal volume of Freund’s complete adjuvant and injected intraperitoneally with 100 μl heat-inactivated *Bordetella pertussis* toxin (Sigma-Aldrich, Poole, UK). The immune response was boosted one week later. Twenty-one days after the initial immunisation, murine AIA was induced by intra-articular (IA) injection of 10 mg/ml mBSA in the right knee joint. Left knee joints were treated as controls by receiving PBS injections.

Twenty hours after arthritis induction, 10 μl of serum free CEM, containing 300,000 MSCs either labelled with or without SiMAG (10 μg/ml) were injected IA into the right knee joint. Control animals were injected with only serum free CEM. Explanation of the experimental groups can be found in Table 1. Upon experiment termination, animals were sacrificed, prepared for MRI and joints collected for histological assessment.

**Table 1:** Explanations of experimental groups

***Group***	***Description***	***Tracking period***
***1 (n=6)***	SiMAG-labelled mMSCs	3 days
***2 (n=5)***	SiMAG-labelled mMSCs	7 days
***3 (n=6)***	Unlabelled mMSCs	3 days
***4 (n=5)***	Unlabelled mMSCs	7 days
***5 (n=6)***	No cells + No Particles (control)	3 days
***6 (n=5)***	No cells + No Particles (control)	7 days

mMSCs, murine mesenchymal stem cells.

Animals were inspected daily for arthritis development by measuring knee joint diameters using a digital micrometer. The difference in joint diameter between the arthritic (right) and non-arthritic control (left) knee in each animal gave a quantitative measure of swelling (in mm). Statistical analysis was performed using unpaired *t*-test.

### Magnetic resonance imaging

#### Evaluating minimum visibility threshold

*In vitro* minimum visibility was investigated by resuspending mMSC labelled with 0, 1, 5 and 10 μg/ml SiMAG at various cell dosages (10^3^, 10^4,^ 10^5^, 3 × 10^5^, 5 × 10^5^) in 2 mg/ml rat tail type I collagen (BD Biosciences, Oxford UK). Samples were MR imaged using a Brucker 2.3 T animal scanner (Nottingham Trent University). The relaxation parameters T_1_ and T_2_^eff^ were estimated for each of the samples. T_2_^eff^ was estimated using a Multi Slice Multi Spin Echo (MSME) imaging sequence and fitted exponential to the envelope of the echoes. The repetition time was set at five seconds and the echo time at the lowest value, 10.173 ms. A matrix size of 256 × 128 gave a spatial resolution of (0.35 × 0.55) mm. T_1_ was estimated using the same sequence with a repetition time varied between 100 and 5,000 ms and fitted exponential to the first echo in the train of each image.

Similarly, the *in vivo* visibility threshold was investigated by injecting 3 × 10^5^ cells labelled with 0, 1, 5 and 10 μg/ml SiMAGIA into both joints of healthy (non- arthritic) 10-week-old BALB/c mice (N = 2) and MR imaged on the same system. In order to improve the contrast around the joints the two imaging sequences chosen were Rapid Acquisition with Refocused Echoes (RARE) and Gradient-echo Fast Imaging (GEFI) for T_1_ and T_2_^eff^, respectively. Sequence parameters were as follows: RARE, matrix size: 256 × 192, spatial resolution: (0.47 × 0.42) mm, repetition time: 4,000 ms, effective echo time: 21.8 ms; GEFI, matrix size: 256 × 256, spatial resolution: (0.47 × 0.47) mm, repetition time: 500 ms, echo time: 4.7 ms, flip angle: 30°.

#### Magnetic resonance imaging tracking

Mice were sacrificed either three or seven days post cell implantation and imaged using a Brucker 2.3 T animal scanner (Nottingham Trent University). Whole body Fat Low Angle Shot (FLASH) and RARE sequences (detailed above) were employed to image day three groups while GEFI and RARE were used for day seven groups to determine the location of the particle-labelled cells. FLASH, matrix size: 256 × 256, spatial resolution: (0.45 × 0.29) mm, repetition time: 1000 ms, echo time: 3.35 ms.

### Histological assessment

Animals were sacrificed at the indicated times after induction of arthritis. Joints were fixed in neutral buffered formal saline and decalcified with formic acid at 4°C before embedding in paraffin. Mid-sagittal serial sections (7 μm thickness) were cut and stained with H & E. For detection of CM-DiI and SiMAG labelled mMSCs, sections were rehydrated through xylene and alcohol, stained with fluorescent dye (4', 6-diamino-2-phenylindole dihydrochloride (DAPI), Sigma-Aldrich), mounted in Hydromount (National Diagnostics, Hessle UK) and imaged using a UV fluorescent microscope (Nikon Eclipse Ti-S). Prussian blue staining was subsequently carried out to identify the presence of SiMAG labelled cells within joint sections while toluidine blue staining was used to assess cartilage damage.

## Results

### Particle uptake

**Figure 1:**
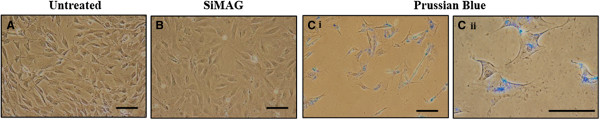
**Evaluation of morphology and SiMAG uptake by mMSCs. A)** Unlabelled mMSCs, **B)** SiMAG-labelled mMSCs, **C)** Prussian blue stain of SiMAG-labelled cells; (i) x 20 magnification, (ii) x 40 magnification. Bars =100 μm. mMSC, murine mesenchymal stem cells.

Particle uptake following a 24 hour passive incubation period of SiMAG with mMSCs was confirmed by Prussian blue staining (Figure 1). Internalised particles were stained blue and were visible within the cell. Intracellular Fe content was measured by ICP as 20 ± 0.04 pg/cell. Cells retained morphology post labelling.

### Evaluating minimum visibility threshold

**Figure 2:**
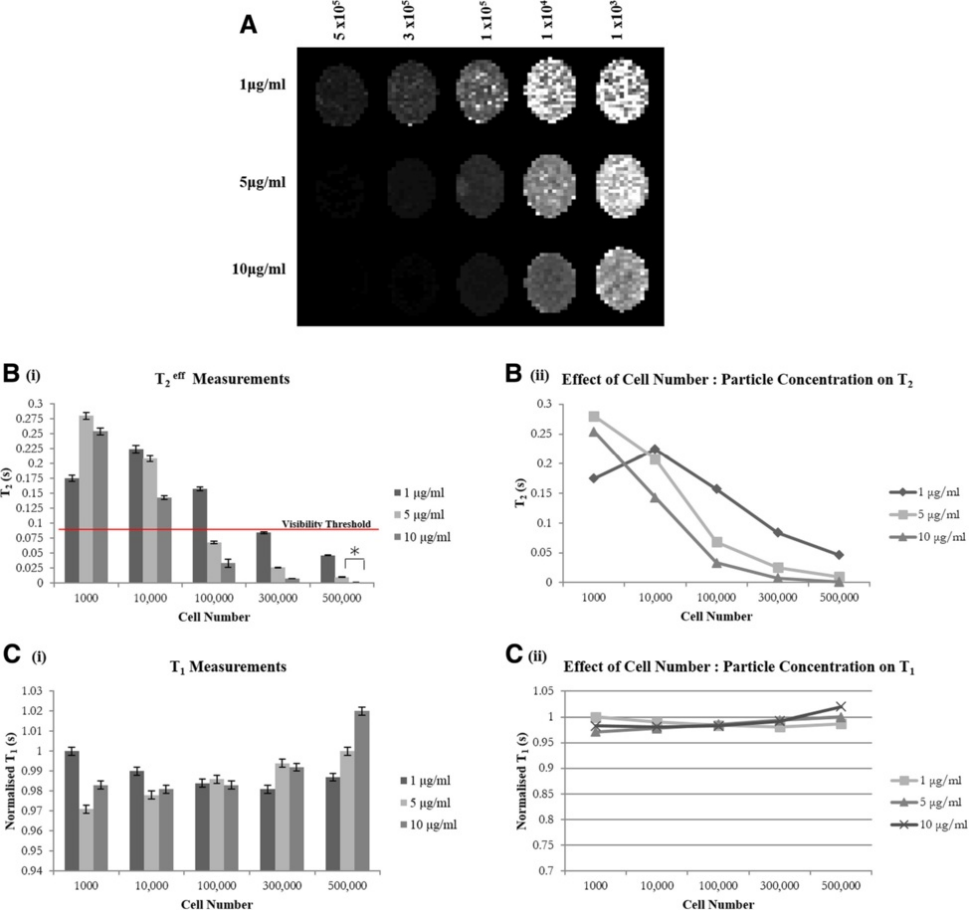
***In vitro *dose response. A)** T_2_^eff^ map for 1, 5 and 10 μg/ml SiMAG-labelled cells ranging in cell doses (10^3^, 10^4^, 10^5^, 3 × 10^5^, 5 × 10^5^). **B)** Bar chart depicting i) T_2_ and ii) normalised T_1_ measurements at each particle concentration (1 to 10 μg/ml) and cell dose (10^3^, 10^4^, 10^5^, 3 × 10^5^, 5×10^5^). Red line indicates MRI visibility threshold implying that any cell: particle combination resulting in a T_2_ less than 0.084 ms can be detected by MRI. **C)** Plot to show correlation between number of cells and i) T_2_^eff^ and ii) normalised T_1_ for three different concentrations (1, 5 and 10 μg/ml) of SiMAG. Data = average ± SD (n = 6). Assume *** significant levels unless otherwise stated (*P* <0.005). *Indicates significant statistical difference in T_2_ between 500,000 cells labelled with either 5 μg/ml or 10 μg/ml (*P* <0.01). MRI, magnetic resonance imaging; T_1_, longitudinal relaxation time; T_2_, transverse relaxation time; T^2eff^, effective transverse relaxation time.

Figure 2 demonstrates the relationship between SiMAG concentration and cell number in terms of MRI signal loss or hypointensity. Hypointense regions of signal voids highlight the presence of SiMAG-labelled cells. As expected, hypointensity increases with cell number and SiMAG concentration (Figure 2A). T_2_^eff^ was found to be shorter for higher numbers of labelled cells with this further decreasing with increasing SiMAG concentration from 1 μg/ml to 10 μg/ml (Figure 2B (i,ii)). MRI detection thresholds were investigated both *in vitro* and *in vivo* and set at a T_2_^eff^ value of 0.084 ms *in vitro*. This corresponded to a minimum SiMAG concentration of 1 μg/ml (300,000 cells) and a minimum cell dose of 100,000 cells (5 and 10 μg/ml). In essence, any cell: particle combination that results in a T_2_^eff^ value of 0.084 ms or below will be visible by MRI. The T_2_^eff^ at 1,000 cells labelled with 1 μg/ml resulted in a lower value than anticipated (0.175 ms). This is likely due to poor distribution of the small number of cells. There is a strong exponential relationship between the number of cells at each of the concentrations tested and T_2_^eff^ whilst no variation was seen with T_1_ (Figure 2C (i, ii)).

**Figure 3:**
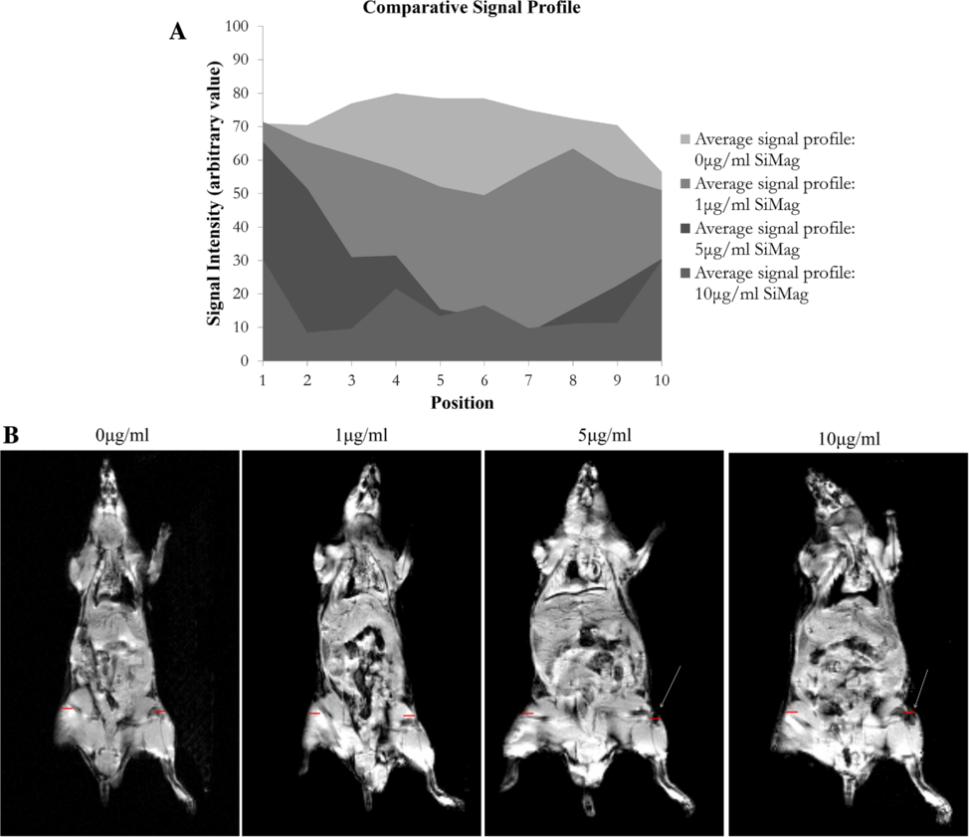
***In vivo *dose response. A)** Signal profile and **B)** corresponding coronal GEFI MR images relating to mMSCs labelled with 0 μg/ml (i), 1 μg/ml (ii), 5 μg/ml (iii) or 10 μg/ml (iv) and implanted within the right knee of each mouse. Red line highlights the area across which SI was measured. GEFI, gradient-echo fast imaging; mMSCs, murine mesenchymal stem cells; MR, magnetic resonance; SI, signal intensity.

*In vivo* dose response was assessed by analysing signal profiles when 300,000 mMSCs labelled with 0, 1, 5 and 10 μg/ml SiMAG were injected within the synovial cavity of a mouse (Figure 3). Signal intensity (SI) across the hypointense region was read, plotted and compared (Figure 3A). A greater loss in signal over a greater area was measured in mice injected with 5 and 10 μg/ml SiMAG, with an overall SI loss of 61% and 78% respectively. A less obvious drop in SI was noticed for mice injected with 1 μg/ml when compared to the control mouse with a SI loss of 20%.

### In vitro assessment of SiMAG labelling on mMSC characterisation, differentiation, viability and proliferation

**Figure 4:**
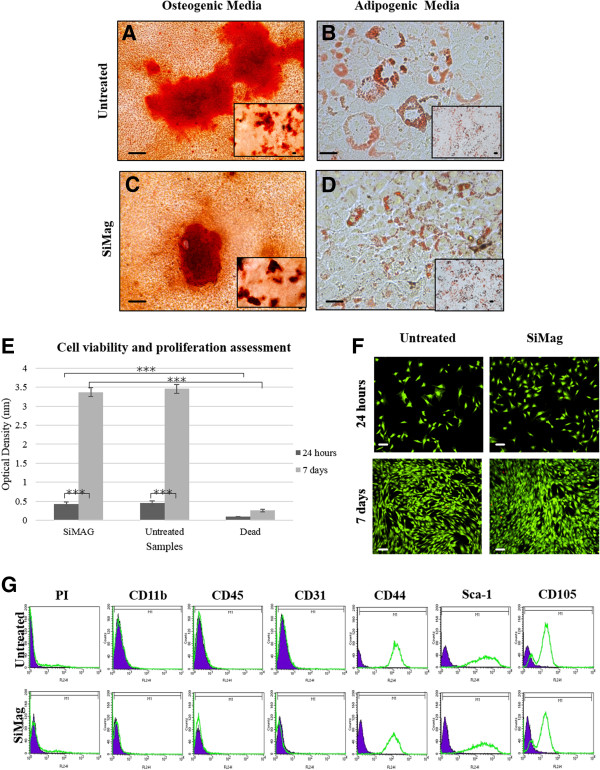
**Immunophenotypic characterization, cell viability, proliferation and differentiation of SiMAG-labelled mMSCS.** mMSCs labelled with **(C, D)** and without **(A, B)** SiMAG underwent successful differentiation down osteogenic **(A, C)** and adipogenic **(B, D)** lineages after 21 days. mMSCs labelled with 10 μg/ml of SiMAG characterised at 24 hours and 7 days by **E)** MTT analysis and **F)** live dead staining. *** Indicates *P* <0.001. Bars = 100 μm. **(G)** SiMAG-labelled and unlabelled mMSCs were negative for hematopoietic markers CD11b and CD45, endothelial cell marker CD31 and positive for CD44, CD105and Sca-1. The expression of every antigen is shown together with their corresponding isotype control. mMSCs, murine mesenchymal stem cells.

mMSCs either labelled with SiMAG (10 μg/ml) or without SiMAG underwent successful differentiation towards osteogenic and adipogenic lineages after 21 days in culture with relevant differentiation media. Calcium deposition was stained positive by Alizarin red in both labelled and unlabelled cells confirming osteogenesis (Figure 4A,C). Adipogenesis was confirmed by the presence of lipid and triglyceride droplets stained positive with oil red O after 21 days for SiMAG-labelled and unlabelled cells (Figure 4B,D). MTT (Figure 4E) and live dead (Figure 4F) analysis revealed no diminished viability and proliferation capacity for SiMAG-labelled cells at either 24 hours or 7 days with no significant differences in metabolic activity between treated and untreated groups at either 24 hours nor 7 days. The significant increase (*P* <0.005) in metabolic activity from 24 hours to 7 days as exhibited by both groups highlights the proliferative capabilities of labelled and unlabelled cells as counter validated by live dead analysis (Figure 4F).

Fluorescence activated cell sorting (FACS) analysis demonstrated that mMSCs were negative for hematopoietic markers CD11b and CD45, the endothelial cell marker CD31 (PECAM), and positive for the mesenchymal markers CD44 and CD105, including the stem cell marker Sca-1. Propidium iodide staining confirmed a cell viability of >95% for both SiMAG-labelled and unlabelled cell populations. Labelling cells with SiMAG did not have an effect on the immunophenotype of mMSC (Figure 4G).

### Rheumatoid arthritis progression (joint swelling)

**Figure 5:**
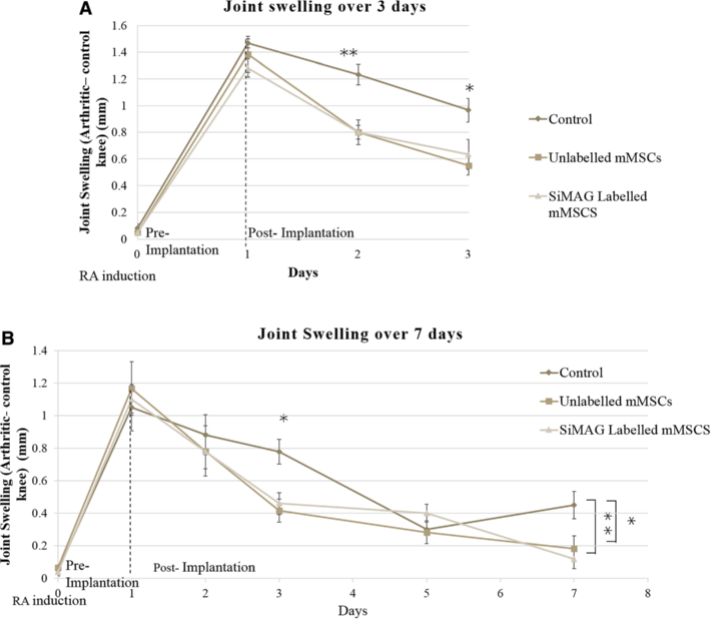
**Joint swelling measurements (mm) as an indication of inflammation and RA progression.** Comparing joint swelling between SiMAG-labelled mMSCs, unlabelled mMSCs and control groups over **A)** three days and **B)** seven days. Data are mean ± SEM for the right knee after subtraction of the left knee control. Significant levels ** indicates *P* <0.01 and * indicates *P <0.05*. mMSCs, murine mesenchymal stem cells; RA, rheumatoid arthritis; SEM, standard error of the mean.

This model (AIA) has previously been used to investigate the therapeutic effects of mMSCs in the arthritic mouse by measuring joint swelling (mm) as a clinical indication of joint inflammation (*Kehoe et al.*). Upon RA induction (day 0), knee swelling (mean ± SEM in mm) increased to approximately 1.5 (three day study) and 1.1 (seven day study) times that of the control knee (left). IA administration of either SiMAG labelled or unlabelled mMSCs on day one resulted in an immediate decrease in joint swelling in both studies with a significant drop in swelling measured on day two in the three day study (SiMAG labeled, 0.8 mm, unlabelled mMSCs 0.8 mm versus control group, 1.23 mm) and day three of the seven day study (SiMAG labelled, 0.46 mm, unlabelled mMSCs 0.45 mm versus control group, 0.78 mm). This trend continued in the three day study (SiMAG labelled, 0.6 mm, unlabelled mMSCs 0.5 mm versus control group, 0.9 mm) and ultimately in the seven day study (SiMAG labelled, 0.12 mm, unlabelled mMSCs 0.18 mm versus control group, 0.45 mm). The joint swelling in the control groups is seen to increase in the seven day study from 0.3 mm on day five to 0.45 mm on day seven (Figure 5).

### *In vivo* tracking – magnetic resonance imaging and histological analysis

**Figure 6:**
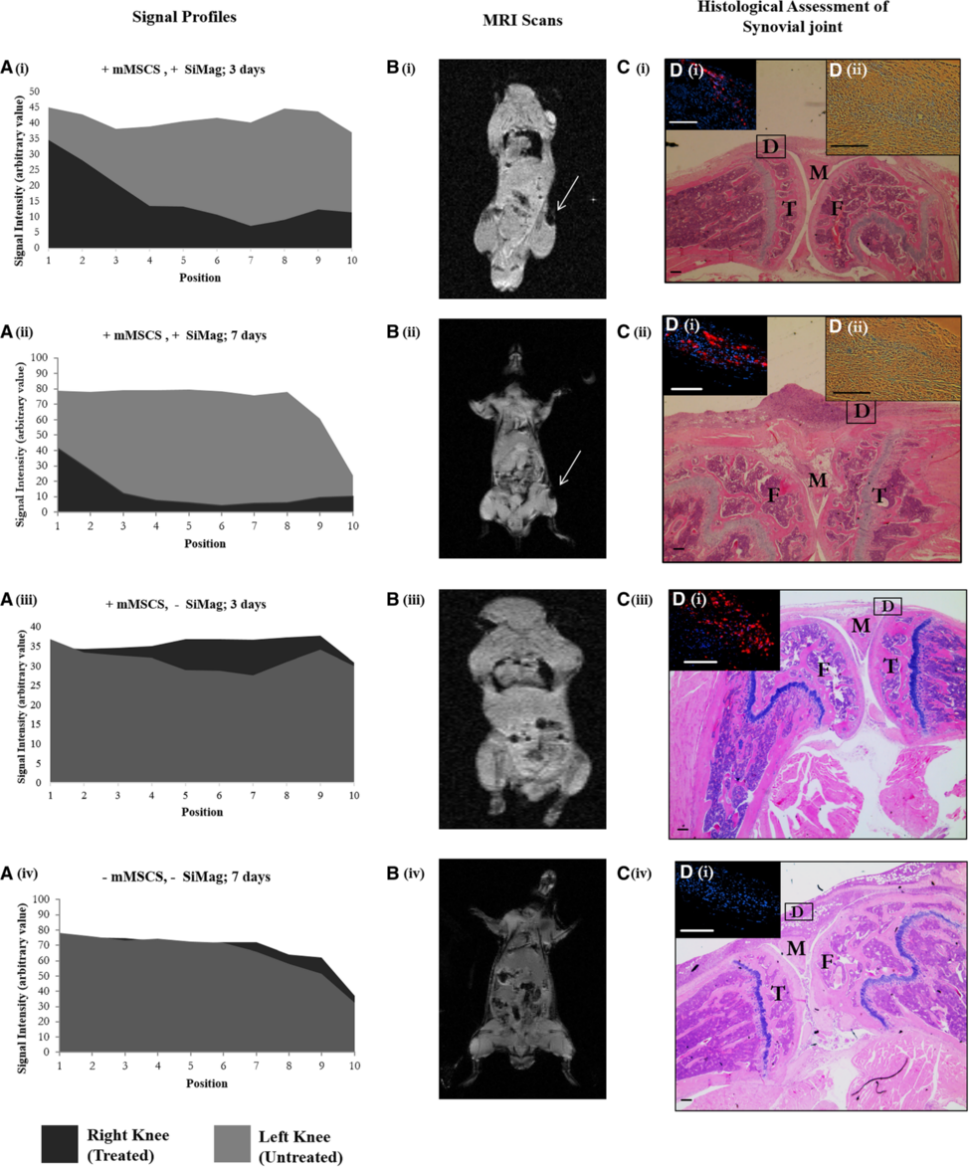
**MRI *in vivo *cell tracking and histological evaluation of synovial joint. A)** Signal profiles, **B)** corresponding coronal FLASH (day 3) and GEFI (day 7) MRI scans, **C)** H & E sections of synovial joint highlighting location of SiMAG labelled cells by D i) CM- DIL (red) and DAPI (blue). Fluorescent analysis and D ii) corresponding Prussian blue stain for i) group 1 (+ mMSCS, + SiMAG; three days, N = 6), ii) group 2 (+ mMSCS, + SiMAG; seven days, N = 5), iii) group 3 (+ mMSCS, - SiMAG; three days, N = 6) and iv) group 6 (− fmMSCS, - SiMAG; seven days, N = 5).T, tibia; F, Femur; M, meniscus. Bars = 100 μm. FLASH, fast low angle shot; GEFI, gradient-echo fast imaging; mMSCs, murine mesenchymal stem cells; MRI, magnetic resonance imaging.

Sacrificed mice were MR imaged on days three and seven using FLASH and GEFI sequences respectively. Hypointense signal voids were observed over the diseased knee (right) in groups 1 and 2 (Figure 6B (i) and 6 (ii)). This represents mMSCs labelled with 10 μg/ml of SiMAG and is shown graphically by plotting signal loss profiles and comparing the signal of the right (diseased) to the left (untreated) joint. It can be noticed that the signal drops greatly over a large area in the right knee of the animal treated with SiMAG-labelled mMSCS as compared to the left knee (Figure 6A (i) and (ii)). This signal loss is attributed to the presence of SiMAG-labelled cells. No signal voids or hypointese regions could be detected in groups 3 and 4 where unlabelled cells were implanted (Figure 6B (iii)) nor in groups 5 and 6 where no cells were implanted at all (Figure 6 (iv)). This is again validated by the signal loss profiles where similar high-signal profiles are shown for both the right (diseased) and left (untreated) joint in (Figure 6A (iii) and (iv)). Immunohistological analysis of tissue sections revealed the presence of fluorescently (DIL and DAPI) labelled cells within the synovial joint, specifically in the lining and sub-lining layers of the synovium, in the region of the patella, and femoral and tibial surfaces of all mice in groups 1 to 4, seven days post implantation (Figure 6C (i to iii) and D i) (i to iii)). No labelled cells were found in control groups (Figure 6C (iv) and D i) (iv)). Further to this, no localisation of implanted cells was observed in other joint tissues. Prussian blue staining successfully revealed the presence of iron (stained blue) in knee sections for groups receiving SiMAG labelled mMSCs (groups 1 and 2) (Figure 6C (i,ii) and D ii) (i,ii)).

**Figure 7:**
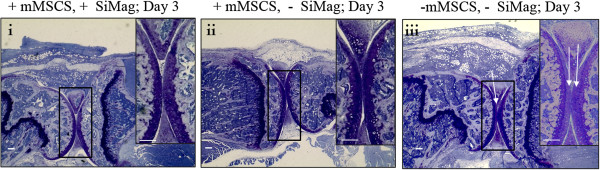
**Toluidine staining to demonstrate cartilage depletion within joint sections for i) group 1 (+ mMSCS, + SiMAG; three days, N = 6), ii) group 3 (+ mMSCS,- SiMAG; three days, N = 6) and iii) group 5 (− mMSCS, - SiMAG; three days, N = 6).** Bars = 100 μm. mMSCs, murine mesenchymal stem cells.

Toluidine blue primarily stains for acidic proteoglycans found in articular cartilage making it an applicable stain to assess cartilage depletion in arthritis. Joint sections highlighted the loss of proteoglycans from the surface of articular cartilage in the control group (Figure 7iii). Cartilage depletion was less pronounced in groups receiving mMSC treatments after three days (groups 1 and 3).

## Discussion

In response to the lack of pharmacological interventions to treat RA, cell therapies have been developed, offering new opportunities in tackling this disease. Stem cells, such as MSCs, have been used to regenerate and restore the function of damaged tissue, such as cartilage [7]. The striking ability of MSCs to migrate toward the site of injury, engraft with surrounding tissues [28] and differentiate into cartilage [29] whilst suppressing the immune system, highlights their applicability as a possible therapy for RA [2]. Despite the vast number of studies demonstrating the potential of MSCs in treating RA, the clinical translation and commercialisation of this therapy is hindered by additional unknown factors [7]. A non-invasive online method of monitoring the success of the therapy in terms of cellular bio distribution [30] and cell survival [31] is key in deciphering the mechanism of repair, evaluating the therapeutic effect, grafting location and ruling out potentially dangerous side effects [17]. This would encourage regulatory approval to be obtained and therapeutic potential optimised for clinical applications [28].

The precedent for labelling cells with magnetic nanoparticles (MNPs) or SPIONS comes from the technology employed in MRI and MRI contrast agents and offers an attractive way of monitoring cells *in vivo*[28,32-34]. SPIONs are essentially composed of a magnetite (Fe_3_O_4_) or maghemite (γ-Fe_2_O_3_) core coated with a biocompatible polymer [35]. The range of particles commercially available is vast with particles ranging in size from the nanometer to the micron scale [17,34] and differing in polymer coating and surface chemistries. An extensive range of studies utilising an assortment of MNPs have been used for a variety of tissue engineering studies. There is no clear consensus as to the ideal MNP size, concentration or magnet strength that would yield the greatest contrast *in vivo*. As previously mentioned, 1.5 and 3 T scanners are commonly found in clinical practise today. With this in mind, it is essential to design and optimise imaging and tracking protocols in terms of cell number and particle concentration to these clinically relevant scanners. Particle concentration ranging from 12.5 to 400 μg/ml have been reportedly used in studies focusing on 1.5 T scanners [16,22,28] while 2.8 to 168 μg/ml have been reported in studies using 3 T scanners [16,24,31]. In this study, a particle concentration of 10 μg/ml has been implemented within a 2.3 T scanner highlighting the clinical relevance of our system.

In recent years, a number of studies focusing on the development of strategies to image and track stem cells within the articular joint have emerged. These studies tend to adopt invasive surgical models of osteoarthritis in either rabbits or rats where cells (MSCs or chondrocytes) are labelled with MNPs, seeded onto scaffolds, such as collagen type I gel [25] or agarose [36], and implanted within osteochondral defects. Animals are then monitored over a period ranging from four to twelve weeks while being MR imaged [13,25,36,37]. Particles used in these studies have ranged from very small superparamagnetic iron oxide nanoparticles (11 nm) [25] to Food and Drug Administration approved particles such as Endorem (150 nm) [37] and Feraheme (30 nm) [36]. Contrary to the above group of studies where MNP ranged in size between 11 nm and 150 nm in diameter, *Saldanha et al.* investigated the use of micron sized particles (1.63 μm) as a potential tracking agent to monitor cartilage regeneration. Rabbit MSC were labelled with a commercially available particle and implanted within an *ex vivo* knee model (bovine) and MR imaged using 3 T to monitor articular cartilage regeneration [19].

From these studies, it is evident that tracking studies have primarily focused on the rat or rabbit models where surgical techniques are used to mimic trauma or osteoarthritis. In addition, cells have generally been labelled with a maximum particle size of 150 nm and seeded onto hydrogel scaffolds prior to implantation. The end point of such animal studies is often histological assessment. Animal models, however, also allow for the investigation of functional outcomes, such as the presence of inflammation. Very few stem cell related studies actually monitor histopathological features while assessing functional outcomes and tracking cells *in vivo*.

Particle uptake is quantified by measuring the total intracellular iron (Fe) content. This value is dependent upon on a number of factors including particle size, surface charge, labelling protocol and the rate of cell proliferation [25,38]. In this study, a 24 hour serum-free passive incubation method was applied. This method tends to be limited to cells with a high degree of phagocytosis, a property not exhibited by stem cells [39]. The addition of transfection agents (TA) may thus be required to stimulate phagocytosis [24]. Studies by Hill *et al.* and Saldanha *et al*. have demonstrated the efficient uptake of micron scale particles by MSCs via passive incubation without the use of TA [19,23]. In this study, 1 μm, commercially available particles, SiMAG, have been used and resulted in a labelling efficiency of 80% with a total iron content of 20 pg of Fe/cell. This value is comparable to other studies where internal Fe content ranged from 1 to 30 pg of Fe/cell compared to a value of 0.1 pg of Fe/cell for unlabelled cells [18,20].

Internalised Fe disrupts the local magnetic field causing a shortening of T_2_ and T_2_*, thereby allowing SPION-labelled cells to be visualised as signal voids (blackened marks) or hypointense areas on MRI scans [19-25]. It is essential to establish MRI visibility thresholds in terms of particle concentration and cell number, both *in vivo* and *in vitro* and to appreciate that the detection threshold is affected by magnetic field strength and MRI acquisition parameters [20]. *In vitro* detection threshold was set at a T_2_ of 0.0840 ± 0.002 ms corresponding to a minimal cell dose of 100,000 (5 and 10 μg/ml) and particle concentration of 1 μg/ml (300,000 cells). These are considered highly acceptable values as similar studies by Jing *et al.* reported a minimal cell dose of 5 × 10^5^ MSCs when labelled with 25 μg/ml Feridex and the transfection agent protamine sulphate [16]. As expected, T_2_ was seen to decrease with increasing cell numbers and particle concentrations corresponding to the increasing Fe content. T_1_ values were also measured but little difference in T_1_ was noticed. Detection thresholds were validated *in vivo* by implanting 300,000 cells labelled with 1, 5 and 10 μg/ml into each mouse knee and MR imaged using GEFI T_2_ weighted sequences. Relaxivity measurements could not be taken due to the inhomogeneities associated with biological tissue. Signal intensity across the hypointense regions of the knee were read, plotted and compared. The magnetic susceptibility of an individual SPION results in a blooming artefact which extends beyond the size of the individual particles allowing small injections of particles to be amplified beyond the actual location making for practical identification [20,23]. The effect of particle concentration on the blooming effect is clearly seen here. In mice injected with SiMAG (5 and 10 μg/ml), significant loss in signal (61 and 78%, respectively) was measured over a substantial area; however, this signal loss was experienced over a greater area in the mice injected with 10 μg/ml than in those injected with 5 μg/ml. A less obvious drop in SI was observed for mice injected with 1 μg/ml (20%) when compared to the control mice.

Toxicity and safety is a major concern in the implementation of SPIONs in any cell-based therapy [34]. It thus becomes necessary to investigate the viability, proliferation and differentiation potential of SiMAG-labelled cells. A vast number of studies have reported that the use of SPIONs in conjunction with stem cells has little or no effect on the proliferation and viability of cells [18,20,25]. SPIONs are considered to be inert and biocompatible given the nature of Fe [18]. Fe is a naturally occurring element in the human body playing an important role in cellular metabolic processes, such as DNA synthesis, oxygen transport and redox reactions [29,34]. The body is therefore adapted for Fe metabolism and thus labelling MSCs with SPIONs is not likely to affect the biological properties of cells [29]. In high quantities, however, Fe can possibly impair cell viability by damaging cell membranes, proteins and DNA [28,29]. Therefore, it is important to obtain a balance between Fe incorporation for the required role and cell function [28]. Cell viability assays revealed that cell viability was maintained in SiMAG-labelled cell populations up to seven days post labelling with no significant effect on cell proliferation capacity either. Furthermore, FACS data demonstrated similar profiles for SiMAG-labelled cells and unlabelled cells implying that the presence of SiMAG does not alter the MSC cell surface markers.

Currently, there is a debate regarding the differentiation ability of cells once they have been labelled with SPIONS [25]. Generally, studies have found that the osteogenic and apidogenic potential of MSCs was maintained post SPION labelling whilst there are conflicting reviews reported for the differentiation of SPION labelled MSCs to chondrocytes [25,29]. Studies have shown that stem cells labelled with Feridex (an FDA approved contrast) inhibited their differentiation towards the chondrogenic lineage but this was not the case for osteogenic and adipogenic lineages [25,28,40]. Kostura *et al.* found that the presence of Feridex interfered with the signalling pathway responsible for driving chondrogenic differentiation [40]. In contrast, Jasmin *et al.* reported that MCSs labelled with Feridex underwent successful differentiation down all three lineages (adipogenesis, chondrogenesis and osteogenesis) [30]. In a study by Henning *et al.*, it was shown that differentiation to chondrocytes was dose dependent which may explain the contrasting results [29]. Henning also suggested that the use of a TA could mitigate this effect by encouraging the internalisation of particles via an alternative mechanism and further compartmentalization which might cause less interaction with differentiation linked intracellular substrates [29]. We have not been able to demonstrate successfully the differentiation of BALB/c mMSCs to chondrocytes in either labelled or unlabelled cells. In a study by Chamberlain *et al.* BALB/c derived mMSCs were shown to differentiate down osteogenic and adipogenic, but not chondrogenic, lineages [26]. We cannot, therefore, contribute the lack of chondrogenic potential to the presence of SiMAG but to properties of the cells themselves. Importantly, we have shown that mMSCs labelled with 10 ug/ml of SiMAG retain their capability to differentiate successfully down osteogenic and adipogenic lineages.

The chosen model bears numerous similarities to the human mode of the disease and is thus suitable for predicating the efficacy and appropriateness of this therapy in humans [41]. It must be noted that the rodent version of this disease progresses at a faster rate than does the human version. Therefore, any interventions are monitored by assessing acute inflammation (macrophage, lymphocyte and neutrophil infiltration) in terms of joint swelling [3,41]. As expected, a significant decrease in joint swelling was measured upon mMSC administration. In a similar way, the administration of SiMAG-labelled mMSC also resulted in a significant decrease in joint swelling with no statistical difference between the mMSC groups. This suggests that the immunomodulating properties of mMSC labelled with SiMAG are maintained. Biological variation between mice within groups accounts for the conflicting rates of joint swelling progression between the two studies where a significant drop in joint swelling was found on day two for the three day study but only on day three for the seven day study. A limitation of this study in terms of assessing the therapeutic effects of mMSCs in treating RA would be the low N numbers of the study.

The therapeutic effects of mMSCs in the same model of RA were assessed by *Kehoe et al.* (paper under review). This study concluded that mMSCs are therapeutic when injected into the joints of mice with AIA as demonstrated by reduced levels of cartilage destruction. Toluidine blue staining demonstrated far less cartilage depletion in groups receiving either SiMAG labelled or unlabelled mMSC when compared to control groups. This suggests that the therapeutic potential of mMSCs is not affected by SiMAG labelling. Although the mechanism of action is unknown, it is unlikely that the implanted cells are differentiating into chondrocytes and repopulating the damaged area as no fluorescently labelled cells could be found within the articular cartilage, corroborating findings by *Kehoe et al*. It has been suggested by *Kehoe et al.* that the mechanism of repair is attributed to a paracrine effect whereby the early MSC treatment acts to prevent proteoglycan loss via the secretion of factors influencing the activity of ADAMTS enzymes, an enzyme responsible for the cleaving of aggrecan (an abundant proteoglycan in articular cartilage).

In terms of imaging and tracking, the blooming effect of the particles and the application of T_2_ weighted sequences (GEFI/FLASH) made for simple identification of the implanted SiMAG-labelled cell population and the location was easily identified as the synovial cavity up to seven days post implantation. This was shown graphically by comparing the SI of joints treated with SiMAG-labelled cells to unlabelled cells and to untreated knees, where a significant loss of signal was measured, further highlighting the position of cells. This was further verified via histological analysis where DiI labelled cells were clearly seen within the synovial joint up to seven days post implantation.

The use of MRI has a dual purpose. Not only can it be used to image and track implanted cells, but its ability to distinguish between cartilage and bone [3] can be used to assess the defect and determine the extent of cartilage repair. The amount of fill in the image could reflect the extent of repair while comparing the signal of the new graft with surrounding tissue could indicate the maturity of the graft [42]. As mentioned previously, the blooming effect of the cells allows for the simple identification of cells *in vivo.* However, when applied to such a small joint, it is impossible to analyse tissue repair as it blocks all surface anatomy.

## Conclusions

Although this study has not assessed the extent of cartilage repair, there is no reason to believe that the presence of SiMAG will affect the repair process corroborating results seen in a similar study from *Kehoe et al.* (under review).

Regenerative medicine and tissue engineering thrive on the cross collaboration of multiple fields to aid in the application of stem cell based therapies. This often involves the development and implementation of enabling technologies (as described in the manuscript) in line with regulatory bodies to bring stem cell based therapies closer to the clinic. Feridex and Endorem are examples of FDA approved, iron-based MRI contrast agents that have previously been used in conjunction with TAs in similar studies. Unfortunately, these particles have recently been taken off the market [34] with no other suitable replacements. Therefore, the need to identify and implement a new particle is necessary. The ideal labelling agent should allow for the repetitive, non-destructive and non-invasive long term tracking of implanted cells while maintaining cellular function at a realistic dose within a clinically relevant system [22]. SiMAG has been shown successfully to maintain cellular function at realistic doses within a clinically relevant system; however, its application in long term tracking has yet to be investigated. This manuscript thus highlights the potential of SiMAG to be used and adapted as a suitable agent for the imaging and tracking of cell populations via MR imaging in the clinical translation of a wide range of cell therapies.

## Abbreviations

ADAMTS: A disintegrin and metalloproteinase with thrombospondin motifs; AIA: Antigen-induced arthritis; BSA: Bovine serum albumin; CEM: Complete expansion media; CIM: Cell isolation media; DMSO: Dimethyl sulphoxide; FACS: *Fluorescence activated cell sorting*; FBS: Fetal bovine serum; FDA: Food and Drug Administration; FLASH: Fast low angle shot; GEFI: Gradient-echo fast imaging; H & E: Haematoxylin and eosin; HLA-G: Human leukocyte antigen G; IA: Intra-articular; ICP-OES: Inductively coupled plasma optical emission spectrometry; IDO: Indoleamine 2,3-dioxygenase; IL: Interleukin; IMDM: Iscove modified Dulbecco medium; mMSC: murine mesenchymal stem cells; MNP: magnetic nanoparticles; MRI: Magnetic resonance imaging; PBS: Phosphate-buffered saline; RA: Rheumatoid arthritis; RARE: Rapid acquisition relaxation–enhanced; SI: Signal intensity; SPIONs: Superparamagentic iron oxide nanoparticles; TA: Transfection agent; T1: Longitudinal relaxation time; T2: Transverse relaxation time; T2eff: Effective transverse relaxation time; TGF-β: Transforming growth factor beta.

## Competing interests

The authors declare that they have no competing interests.

## Authors’ contributions

Project conception: AEH and OK. Experimental design: HM, OK and AEH. Animal work: OK. *In vitro* cell work, optimisation and data analysis: HM. MRI scanning and analysis: RM and HM. Manuscript written by HM and revised by all authors. All authors have read and approved the final manuscript.
